# Leveraging the synergy between anti-angiogenic therapy and immune checkpoint inhibitors to treat digestive system cancers

**DOI:** 10.3389/fimmu.2024.1487610

**Published:** 2024-12-03

**Authors:** Qinlan Xu, Dong Shao

**Affiliations:** Department of Gastroenterology, The Third Affiliated Hospital of Soochow University, Changzhou, Jiangsu, China

**Keywords:** digestive system cancer, anti-angiogenic therapy, immune checkpoint blockade, tumor microenvironment, immunotherapy, vessel normalization

## Abstract

The response rates to immunotherapy vary widely depending on the type of cancer and the specific treatment used and can be disappointingly low for many solid tumors. Fortunately, due to their complementary mechanisms of action, immunotherapy and anti-angiogenic therapy have synergistic effects in cancer treatment. By normalizing the tumor vasculature, anti-angiogenic therapy can improve blood flow and oxygenation to facilitate better immune cell infiltration into the tumor and enhance the effectiveness of immunotherapy. It also reduces immunosuppressive factors and enhances immune activation, to create a more favorable environment for immune cells to attack the tumor. Their combination leverages the strengths of both therapies to enhance anti-tumor effects and improve patient outcomes. This review discusses the vasculature-immunity crosstalk in the tumor microenvironment and summarizes the latest advances in combining anti-angiogenic therapy and immune checkpoint inhibitors to treat digestive system tumors.

## 1 Introduction

Tumor immunotherapy harnesses the body’s immune system to fight cancer and is a transformative approach to cancer treatment (1). It is at the forefront of cancer medicine, providing new hope for patients and reshaping the therapeutic landscape. Immune checkpoint inhibitors (ICIs) form a key branch in the field of tumor immunotherapy, and as of mid-2023, eleven ICIs have been approved by the FDA for treating 20 cancer types. To avoid being attacked by the immune system, cancer cells make use of immune checkpoint proteins (e.g., CTLA-4, PD-1, PD-L1) that maintain self-tolerance and prevent autoimmunity (2). ICIs can block these checkpoint proteins, to thus release the brakes on the immune system and allow T cells to recognize and kill cancer cells (3). ICIs have shown broad bioactivity and lasting effects in treating a wide range of cancers, including melanoma, lung cancer, kidney cancer, bladder cancer, head and neck cancers, etc., thus vastly improving the standard of care (4, 5). However, due to the complexity of the immune environment, it is hard to predict how patients respond to ICIs before deploying the treatment, and there is no single biomarker that can offer satisfactory explanation (6, 7). Treatment-related toxicities are also intricate, since ICIs can over-activate the immune system and cause immune-related adverse events ranging from mild skin rashes to potentially life-threatening myocarditis (8).

The vascularization in tumors, also known as tumor angiogenesis, is generally considered unwanted because it facilitates tumor growth and survival, promotes metastasis, impairs drug delivery, exacerbates hypoxia and acidosis, and causes immune suppression (9, 10). Controlling or inhibiting tumor vascularization is a key strategy in cancer therapy (11). The angiogenic programming of tumors is a multidimensional process involving many cell types, signaling molecules, and microenvironmental factors (12). Tumor cells and other cells within the tumor microenvironment (TME) secrete various angiogenic factors, such as the vascular endothelial growth factor (VEGF), fibroblast growth factors (FGFs), platelet-derived growth factors (PDGFs), etc. These factors, upon binding to their receptors, activate endothelial cells (ECs) to cause morphological changes in the basement membrane and the surrounding extracellular matrix (ECM), thereby promoting the formation of new blood vessels. The expression of these angiogenic growth factors is strengthened by tumor-driven hypoxia, which is crucial for tumor survival and proliferation (11, 13). Anti-angiogenic therapy restricts tumor growth and metastasis by inhibiting the formation of new blood vessels, primarily by targeting molecular pathways critical to angiogenesis. The key targets in anti-angiogenic therapy include VEGF and its receptors (e.g., VEGFR-1 and VEGFR-2), placental growth factor (PLGF), and various other pro-angiogenic mediators (14). Oguntade et al. referred to anti-angiogenesis as “the magic bullet” in cancer therapeutics but also pointed out problems such as tumor resistance and cardiovascular toxicity, and significant interindividual variability has also been reported (15).

Combination therapies that use ICIs and anti-angiogenic agents represent a promising approach in cancer treatment (16, 17). The rationale is that anti-angiogenic therapy can normalize the TME by targeting the tumor vasculature, which improves the anti-tumor immunity and overcomes the resistance to ICIs, and ICIs can then reactivate the immune system for it to recognize and attack cancer cells (18). Early preclinical studies showed that the TME becomes less immunosuppressive and more receptive to immunotherapy upon anti-angiogenic therapy, and in clinical practice, the combination of anti-angiogenic agents and ICIs has been approved for certain cancer types (16). For instance, the combination of bevacizumab (anti-VEGF) and atezolizumab (anti-PD-L1) has been established in lung cancer in combination with chemotherapy as part of the IMpower150 regimen (19). The combination of anti-angiogenic therapy and ICIs is a dynamic and rapidly evolving field, with ongoing research aimed at optimizing these therapies for better patient outcomes. Studies are focusing on understanding the mechanisms of resistance to anti-angiogenic agents, assessing the role of the TME, and identifying biomarkers that can predict the response to therapies. The future of this combination therapy looks promising as it continues to be refined and tailored to individual patient needs and tumor characteristics (20).

This article reviews the advances over the past five years in using ICIs and anti-angiogenic therapies to treat gastrointestinal cancers. It includes ongoing and notable clinical trials that have published results since 2019 and excludes failed and repetitive trials. With a focus on the crosstalk between vasculature and immunity, this work first summarizes the latest advances in preclinical research on the combined therapeutic strategies involving anti-angiogenesis and tumor immunotherapy. It then highlights major clinical breakthroughs achieved through various combination therapies to provide enhanced and personalized treatment options for patients with digestive system cancers, thus demonstrating the effectiveness of integrating anti-angiogenic therapy with ICIs.

## 2 Key signaling molecules in tumor angiogenesis

### 2.1 VEGF

**Figure 1:**
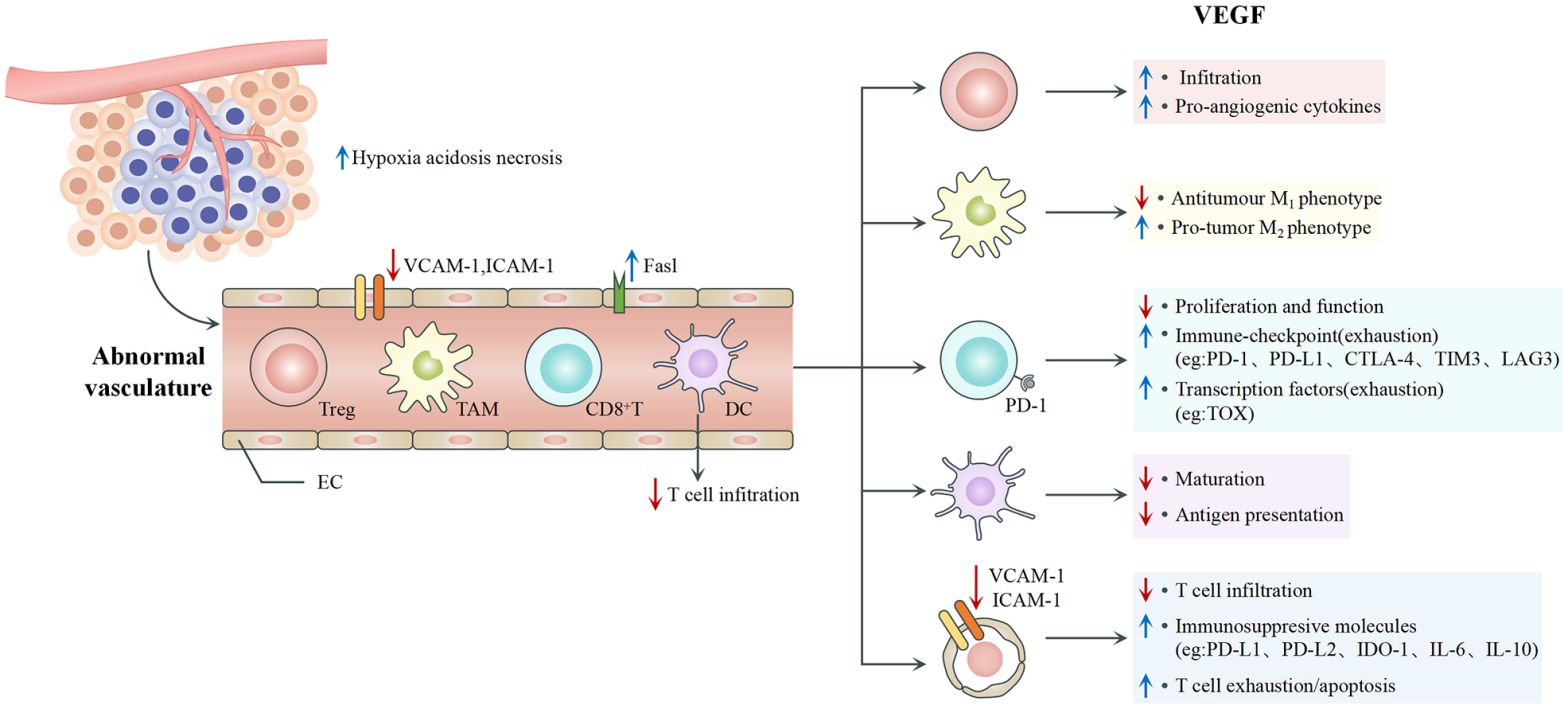
VEGF and angiogenesis.

VEGF is the predominant angiogenic factor expressed in solid tumors. It is a key mediator in the TME and significantly impacts immune cell behavior, tumor vascularization, and cancer progression (**Figure 1**). It mediates tumor angiogenesis by binding to the VEGF receptors (VEGFRs) on ECs, activating downstream signals that promote the proliferation and migration of cells that are needed to form new blood vessels (21). The biological effects of VEGF are mediated mainly through two receptor tyrosine kinases (RTKs), VEGFR-1 and VEGFR-2, which differ substantially in their signaling properties (22). The structure of VEGFRs consists of an extracellular region with seven immunoglobulin-like domains, a transmembrane domain, and an intracellular tyrosine kinase domain (23). Both VEGFR-1 and VEGFR-2 are expressed in vascular endothelial cells, where they work in tandem to regulate angiogenesis. Despite the higher binding affinity of VEGF for VEGFR-1, VEGFR-2 is considered the primary signaling receptor, as most biological effects are mediated through the interaction between VEGF and VEGFR-2 (23, 24). Because of its strong tyrosine kinase activity and pro-angiogenic effects, VEGFR-2 is central to angiogenesis. In contrast, VEGFR-1 has much lower tyrosine kinase activity and minimal ligand-dependent autophosphorylation. It primarily functions as a “decoy receptor” to restrict VEGF binding to VEGFR-2 and thus negatively regulate angiogenesis (24, 25). Additionally, VEGF-B and PLGF bind exclusively to VEGFR-1, and they contribute significantly to pathological angiogenesis and inflammation. The activation of VEGFR-1 further facilitates the recruitment of tumor-associated macrophages (TAMs), which is essential to tumor immune evasion mechanisms within the TME (24, 26).

It can indirectly affect the phenotype and function of immune cells, as VEGFRs are expressed on many immune cells (27). For example, it inhibits the maturation of dendritic cells (DCs) and binds to the receptors on the surface of monocytes to stop them from differentiating into DCs (21, 28, 29). It upregulates the expression of PD-L1 on the surface of DCs to weaken their antigen presentation. High levels of VEGF promote the recruitment and proliferation of Tregs, myeloid-derived suppressor cells (MDSCs), and M2-type TAMs, which collectively suppress anti-tumor immune responses and cause immune evasion (6). VEGF can inhibit the expression of intercellular adhesion molecule 1 (ICAM-1) and vascular cell adhesion molecule 1 (VCAM-1) through VEGFR, thereby suppressing T cell infiltration into tumors (30). Under the influence of tumor-derived factors such as VEGF, ECs can selectively upregulate inhibitory receptors involved in T-cell activation, including PD-L1, PD-L2, IDO-1, IL-6, and IL-10 (31). It can induce the expression of inhibitory receptors on T cells, such as PD-1, CTLA-4, TIM-3, and LAG-3, leading to T cell exhaustion and reduced cytotoxic activity (32). In addition, it can enhance the production of the transcription factor TOX in T cells, triggering a distinct transcriptional program associated with cellular exhaustion (33). It is crucial in creating an immunosuppressive TME favoring tumor growth and evasion from immune-mediated destruction (34). The inhibition of VEGFR activity has been hypothesized to inhibit the liver metastasis of colon cancer, which highlights the critical role of VEGF in cancer progression (35).

### 2.2 FGFs

FGFs play a multifaceted role in the TME (36). They are required for efficient tumor angiogenesis. They stimulate the proliferation, migration, and differentiation of ECs, which are essential steps in the formation of new blood vessels within tumors (37). In digestive system cancers, such as gastric and colorectal cancers, FGFs contribute to the redefinition of the TME by promoting angiogenesis, thereby supporting tumor growth and metastasis (38).

The aberrant signaling of FGFs can directly promote the proliferation and survival of cancer cells. For example, the amplification of FGF receptor 2 (FGFR2), which is less frequent than the amplification of FGF receptor 1 (FGFR1) across cancer types, is often reported in patients with gastric-esophageal junction adenocarcinoma (39). FGF2 has been shown to alter macrophage polarization, shifting TAMs toward a pro-tumorigenic M2-like phenotype (40). This shift can suppress the host’s adaptive immune response, primarily by dampening the activity of CD8^+^ T lymphocytes. The expression levels of FGF receptors (FGFRs), particularly FGFR1 and FGFR2, significantly differ between carcinoma and para-carcinoma tissues in colon, gastric, and esophageal cancers, implicating their role in the susceptibility and progression of these cancers (41).

### 2.3 PDGFs

PDGFs are integral to the complex interplay between immune cells, vascular cells, and cancer cells within the TME. In the context of tumors, PDGF signaling generates abnormal, leaky vessels that contribute to a heterogeneous and hypoxic TME, promoting further tumor growth and metastasis (42). PDGFs exert their angiogenic effect both directly through capillary ECs and indirectly through the recruitment of supporting pericytes that reinforce the walls of the newly formed vessels (43). They can influence macrophage polarization, promoting a shift toward a pro-tumorigenic M2 phenotype (44), which reduces the anti-tumor activity of T cells and NK cells to facilitate tumor growth and progression (45, 46). Their interactions with stromal cells and fibroblasts also foster a supportive niche for tumor cells, enhancing their invasive and metastatic potential (42). The PDGFs receptors (PDGFRs) regulate angiogenesis by promoting vascular maturation, recruiting perivascular cells, and upregulating VEGF expression (43, 47).

In digestive system cancers, such as colorectal cancer, PDGFs/PDGFRs have been implicated in carcinogenesis and may serve as potential targets for diagnosis, prognosis, and therapy (48). Recent studies have highlighted the role of PDGFs in gastrointestinal diseases (49), including their connection to pathological disorders that can lead to cancer, and novel therapies targeting PDGFs are being explored to improve the outcomes for patients with advanced gastric cancer (50).

### 2.4 TGF-β

TGF-β signaling promotes tumor vasculature by enhancing the association between pericytes and ECs, which is crucial for the stability and functionality of blood vessels (51). It supports cancer growth and progression by activating tumor angiogenesis and cancer-associated fibroblasts, enabling the tumor to evade inhibitory immune responses (52). It induces VEGF expression via the Smad3-dependent signaling pathway in tumor cells and stromal cells, such as macrophages, thereby stimulating angiogenesis (53). In colorectal cancer, TGF-β is a master regulator of the epithelial-to-mesenchymal transition, which is a critical step in metastasis, and elevated levels of TGF-β are correlated with tumor progression and immunosuppression (54). Villalba et al. identified a subtype of colorectal cancer with a mesenchymal and aggressive phenotype, with TGF-β as a hub gene of this signature (55).

### 2.5 Angiopoietins

Angiopoietins are a family of vascular growth factors that are pivotal in angiogenesis, and angiopoietin 1 (Ang1) and angiopoietin 2 (Ang2) have antagonistic functions in the context of tumor vascularization (56). Ang1 generally acts to stabilize blood vessels and maintain vascular quiescence. In contrast, Ang2 is often upregulated in the TME and can function to destabilize blood vessels, which can lead to a more aggressive tumor phenotype (13). While Ang1 promotes vessel maturation and the survival of ECs, Ang2 can disrupt the connections between ECs and perivascular cells, promoting vascular regression or sprouting depending on the presence of other factors like VEGF (57). This dynamic balance affects the TME by influencing the delivery of nutrients and immune cells to the tumor, as well as the tumor’s ability to metastasize.

The role of Ang2 in promoting tumor progression is multifaceted. It not only drives angiogenesis but also encourages the infiltration of myeloid cells, which differentiate into stromal cells that facilitate tumor growth and weaken anti-tumor immunity. Ang2 has been shown to contribute to immune suppression by inhibiting the proliferation and differentiation of activated immune effector cells, while recruiting suppressive tumor-associated immune cells such as Tregs, MDSCs, and TAMs. Through integrin signaling, Ang2 can induce the expression of matrix metallopeptidases (MMPs), thus promoting tumor cell invasion and metastasis (58, 59).

### 2.6 IFN-γ

IFN-γ is a critical cytokine in the TME that influences tumor vascularization, immune cell function, and cancer progression. It can induce tumor vascular regression to cause the collapse of tumors like non-hemorrhagic necrosis in ischemia (60). The transfer of the IFN-γ gene into brain tumors results in the secretion of IP-10 and MIG, which can inhibit tumor angiogenesis (61).

IFN-γ has complex roles in the TME. In general, it can polarize TAMs into the M1 phenotype to facilitate vascular remodeling and tumor destruction (62). In colon cancer, ILC1-derived IFN-γ has been shown to regulate macrophage activation and promote the polarization of macrophages toward the M1 phenotype, which is associated with anti-tumor activity (63). It can also inhibit gastric carcinogenesis by inducing the apoptosis of T cells and the autophagy of ECs (64). However, it also has the potential to promote tumor progression under certain conditions. Low doses of IFN-γ have been associated with the acquisition of metastatic properties in tumors, although high doses lead to tumor regression (65).

## 3 Regulation of tumor angiogenesis by immune cells

### 3.1 DCs

DCs are crucial antigen-presenting cells. Different subsets of DCs produce and release various angiogenic factors depending on their activation status and cytokine environment (66, 67). In tumors, the maturation and functions of DCs are regulated by β-defensin (68), VEGF-A (69), MUC-1 (70), and other factors (71). Because immature DCs (iDCs) promote neovascularization through the secretion of paracrine signals, including VEGF, IL-8, and bFGF, encouraging the maturation of DCs can improve the anti-tumor immune response and inhibit tumor angiogenesis (72). Mature DCs (mDCs) are divided into conventional DCs (cDCs) and plasmacytoid DCs (pDCs). While cDCs inhibit tumor angiogenesis and promote M2-type TAM polarization by secreting anti-angiogenic factors such as IL-12, IL-18, CXCL9, CXCL10, and CCL21 (73–75), tumor-associated pDCs induce angiogenesis by secreting TNFα and IL-8 (73).

### 3.2 MDSCs

MDSCs are immune cells that accumulate in tumors and significantly promote tumor growth. They are generally divided into monocytic MDSCs (M-MDSCs) and polymorphonuclear (PMN-MDSCs), which morphologically and phenotypically resemble monocytes and neutrophils, respectively (76, 77). When stimulated by G-CSF, CD11b^+^Gr1^+^ MDSCs directly promote neovascularization through the Bv8 protein and its receptor (78). MDSCs also secrete MMP-9, which regulates the bioavailability of VEGF within tumors and remodels the ECM (79, 80). In fact, MDSCs lacking MMP-9 are incapable of inducing tumor angiogenesis (81). Unlike other immune cells, some MDSCs differentiate into endothelial-like cells expressing CD31 and VEGFR2 and then integrate into the tumor vascular system (76, 81). MDSCs also promote angiogenesis by increasing IL-10 and reducing IL-12 (82, 83).

### 3.3 TAMs

TAMs form the most abundant population of tumor-infiltrating immune cells in the TME and are essential in promoting tumor angiogenesis. They are highly plastic and can be differentiated into the M1 and M2 phenotypes under the influence of various cytokines. While M1 macrophages primarily promote immune functions and attack tumor, M2 macrophages facilitate tissue repair and tumor progression (84, 85). Within the TME, TAMs typically adopt an M2 phenotype, which promotes tumor growth and angiogenesis. However, IFN-γ can reprogram the M2-type TAMs into M1-type TAMs by activating the STAT1 signaling pathway (86).

M1-type TAMs secrete anti-angiogenic factors like IL-12 and TNF-α to inhibit tumor angiogenesis and promote blood vessel maturation, and reducing the vascular density in the tumor suppresses tumor growth and development (85, 87). It is worth noting that the IL-12 secreted by M1-type TAMs can also induce macrophage polarization toward the M1 phenotype, which creates a positive feedback loop to further enhance the anti-angiogenic effect (88). M2-type TAMs promote angiogenesis by releasing pro-angiogenic factors such as VEGFA, epidermal growth factor, and chemotactic factors like CCL2 (89, 90). They also secrete PDGF and TGF-β, which can induce angiogenesis and promote the transition of macrophages from M1 to M2 phenotype.

TAMs can also increase the production of angiogenic factors by inducing pro-inflammatory mediators such as IL-1 and IL-6 (84, 90, 91). TAM-derived MMP-9 can cleave the membrane-bound form of heparin-binding epidermal growth factor to release the soluble, active form, thereby promoting the angiogenic switch (92). Inhibiting CSF1 to deplete macrophages within tumors significantly diminishes the angiogenic potential of breast cancer and tumor burden, but restoring the expression levels of CSF1 can block TAM depletion and enhance the angiogenic potential of tumors (93, 94).

Tie2-expressing monocytes/macrophages (TEMs), a subset of TAMs that express the tyrosine kinase receptor Tie2, are potent enablers of angiogenesis and tissue remodeling. The binding of Ang 2 to Tie2 promotes angiogenesis and abnormal blood vessel formation (95–97).

### 3.4 Tumor-associated neutrophils (TANs)

Neutrophils are essential components of the immune cell repertoire. Within the TME, neutrophils can undergo polarization in response to various cytokine and epigenetic signals, which results in different functional states that can either promote or inhibit tumor progression (98) Neutrophils can be polarized into N1 and N2 phenotypes under the influence of the complex interplay of cytokines, chemokines, and other factors present in the TME. N1 neutrophils are generally considered anti-tumorigenic, and they can kill tumor cells directly and stimulate robust anti-tumor immune responses. In contrast, N2 neutrophils are pro-tumorigenic, and they support tumor growth and metastasis by promoting angiogenesis, suppressing immune responses, and enhancing the ability of cancer cells to invade and migrate.

N2-type TANs can produce MMP-9, remodel the ECM, and release growth factors such as VEGF to promote tumor angiogenesis. In the RIP1-Tag2 transgenic pancreatic neuroendocrine mouse model, neutrophils play a significant role in angiogenesis (98–100). The depletion of neutrophils by administering the Gr1 antibody, a surface marker of neutrophils, reduces the binding between VEGF and its receptor, thereby decreasing tumor angiogenesis (100). In various tumor models, the use of G-CSF to stimulate neutrophils increases the expression levels of Bv8, thereby promoting EC proliferation, tumor migration, and tumor survival (101).

### 3.5 T lymphocytes

Adaptive immune cells can influence tumor angiogenesis by directly changing the biology of ECs and/or indirectly modulating the phenotypes of myeloid cells. Cytotoxic T lymphocytes (CTLs) inhibit tumor angiogenesis through the secretion of IFN-γ, thereby suppressing the proliferation and migration of ECs. They also inhibit tumor angiogenesis by upregulating cytokines that promote pericyte recruitment, including CXCL9, CXCL10, and CXCL11 (60, 102). Among conventional T helper (Th) cells, Th1 cells inhibit angiogenesis, both by producing IFN-γ and by converting M2-TAM into classical pro-inflammatory M1-TAM (103).

In contrast, Th2 cells promote the recruitment of M2-type TAMs and facilitate angiogenesis through the production of IL-4, IL-13, and IL-33 (104, 105). Th17 cells have both protumor and antitumor roles, and its protumor aspect is relevant to tumor angiogenesis (106). They secrete interleukin-17 (IL-17), which promotes tumor angiogenesis by inducing VEGF expression, and IL-17 is correlated with high microvessel density in colorectal cancer patients (107, 108). Th17 cells can also promote angiogenesis via IL-22 (109). Tregs are immunosuppressive and pro-angiogenic, and they indirectly promote angiogenesis by inhibiting the IFN-γ expression in Th1 effector T cells. Notably, Tregs are attracted by hypoxia-induced CCL28, which leads to the expression of VEGFA (110).

### 3.6 B lymphocytes

**Figure 2:**
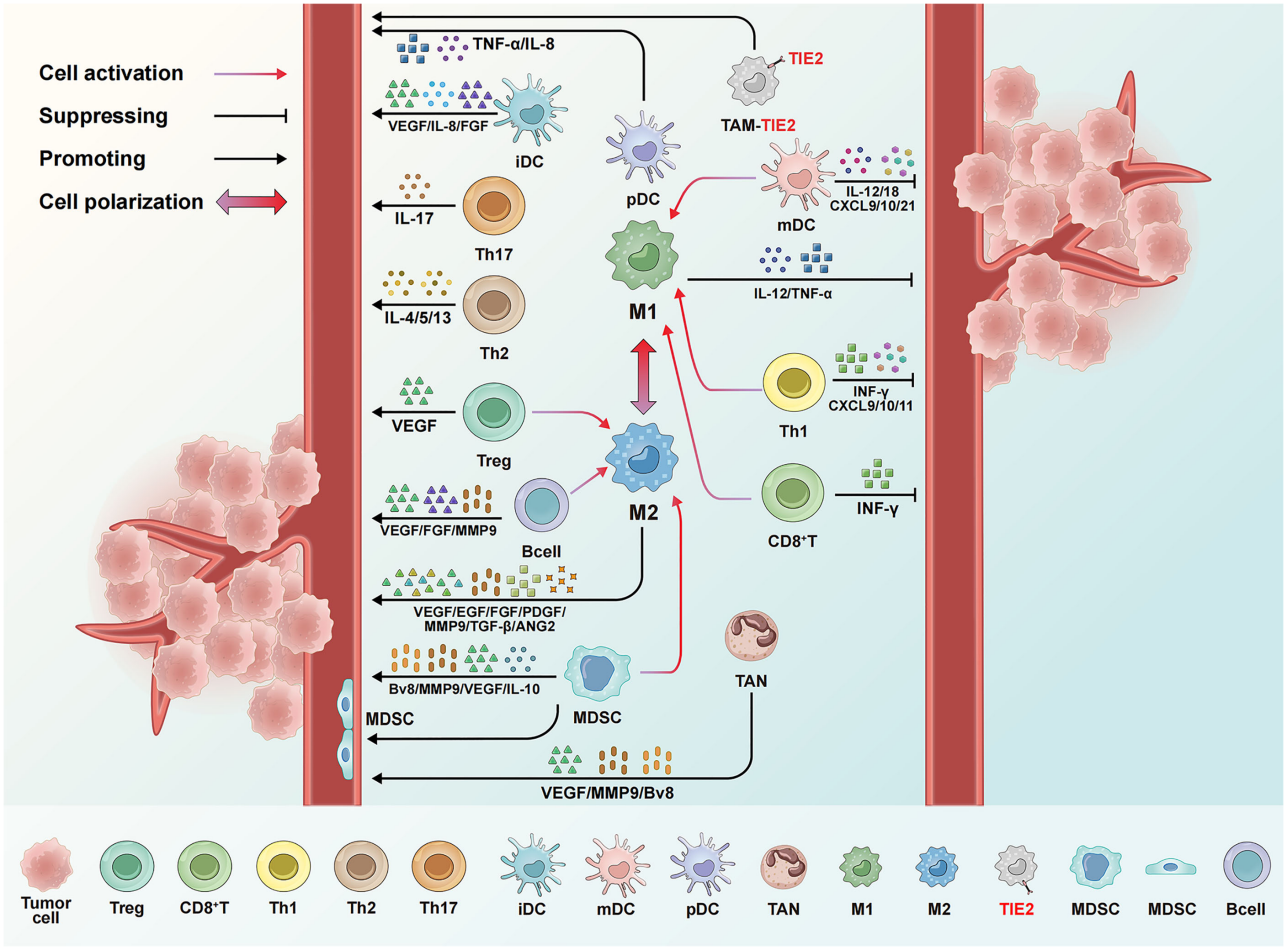
Regulation of tumor angiogenesis by different immune cells. mDCs and M1-type TAMs release IFN-α, IL-12, and other cytokines, alongside chemokines like CXCL9 and CXCL10, to inhibit angiogenesis. CD8^+^T cells and TH1 cells additionally secrete IFN-γ, which suppresses angiogenesis and promotes vascular normalization. Conversely, iDCs, MDSCs, M2-type TAMs, and TEM cells promote angiogenesis through factors like VEGF and IL-10, while Tregs, TH2, and TH17 cells contribute by releasing VEGF and IL-4. Immune cells further regulate angiogenesis through interactions. For instance, mDCs and TH1 cells can polarize macrophages toward the M1 type, whereas MDSCs and Tregs reprogram TAMs toward the M2 type. Some MDSCs can even differentiate into endothelial-like cells and incorporate into the tumor vasculature.

B cells enhance EC function in a STAT3-dependent manner by expressing VEGFA, FGF2, and MMP-9, thereby promoting tumor angiogenesis (111). B cell antibody-antigen complexes induce the production of multiple cytokines, thereby promoting tumor angiogenesis (112). In a transgenic mouse model of HPV16-driven cutaneous carcinoma, the IgG produced by B cells deposits in malignant precancerous skin lesions, which recruits and activates pro-angiogenic factors and polarizes M2-type TAMs, thus driving the onset of skin cancer (113). **Figure 2** summarizes the regulation of tumor angiogenesis by different immune cells.

## 4 Tumor vasculature and immune suppression

### 4.1 Construction of the tumor vasculature system

**Figure 3:**
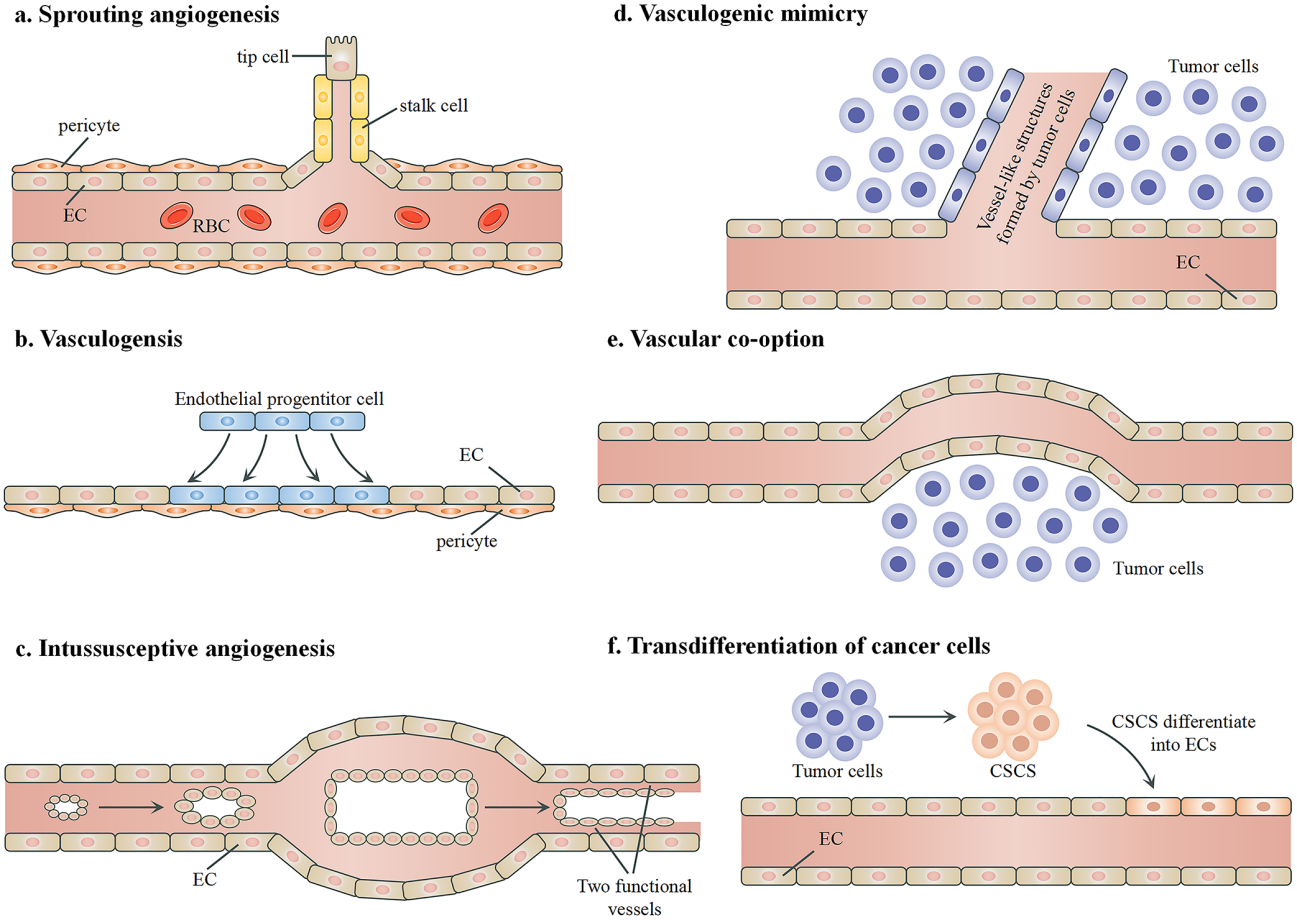
Mechanisms of angiogenesis in tumors. **(A)** Sprouting angiogenesis, during which a new vessel branches from an existing vessel. The key biological process involves the balance between the formation of “tip” and “stalk” endothelial cells. **(B)** Vasculogenesis, during which new blood vessels are generated by endothelial progenitor cells to form networks without the presence of pre-existing vessels. **(C)** Intussusceptive angiogenesis, which involves the formation of a double lumen and the split of an existing vessel into two new functional blood vessels. **(D)** Vascular mimicry. Tumor cells form vessel-like structures independently of endothelial cells to facilitate blood flow. **(E)** Vessel co-option. Tumor cells utilize existing blood vessels for their blood supply. **(F)** Transdifferentiation. Certain cancer stem cells differentiate into endothelial-like cells and integrate into the vascular structure to support the tumor’s blood supply. RBCs, red blood cells; ECs, endothelial cells; CSCs, cancer stem cells.

Angiogenesis in tumors is not limited to the creation of new blood vessels but also includes the utilization and modification of existing vessels and even the transformation of tumor cells to mimic or become vascular cells. These mechanisms are critical for tumor growth and survival and are targets of therapeutic intervention. **Figure 3** summarizes the mechanisms through which tumors develop their vasculature. Like normal tissues, tumors may use conventional angiogenic mechanisms including sprouting angiogenesis, vasculogenesis, and intussusceptive angiogenesis. Sprouting angiogenesis is the primary method of forming blood vessels and entails creating new vessels from existing ones (114). Vasculogenesis involves the transformation of endothelial progenitor cells into ECs to create the initial vascular network (115, 116). Intussusceptive angiogenesis, also known as splitting angiogenesis, refers to the process in which an existing vessel splits into two, although its precise molecular mechanisms are still not completely understood (117). Tumors may also resort to some angiogenic mechanisms that are not found in healthy tissues, including vasculogenic mimicry, vessel co-option, and transdifferentiation. Vasculogenic mimicry is often associated with aggressive and metastatic cancer, and it is used by tumor cells to form vasculature-like structures independently of ECs (118, 119). Through vascular co-option, tumors hijack existing blood vessels in the adjacent healthy tissue to sustain their own growth and metastasis, thereby bypassing the need to generate new blood vessels (120). Transdifferentiation refers to the process where tumor cells transform into endothelial-like cells to contribute to the formation of blood vessel structures within the tumor (121). Understanding the interaction or competition among these mechanisms is crucial for designing and developing novel therapeutic approaches targeting tumor vascularization (122).

### 4.2 Tumor angiogenesis and immunosuppressive microenvironment

Due to a continuous imbalance of angiogenic factors and inhibitors, the tumor vasculature differs significantly from that of normal tissues in both structure and function. For example, tumor blood vessels are often immature and deficient in pericytes, and their high permeability can lead to increased interstitial pressure and the leakage of plasma proteins (123, 124). The turnover rate of ECs in tumor vessels can be significantly higher than in normal tissues. The blood flow within tumor vessels can be erratic and uneven, which creates areas of hypoxia and acidosis within the tumor. In addition, whereas normal blood vessels typically possess an organized hierarchical structure in which arteries, capillaries, and veins are clearly distinguished, the tumor vasculature lacks the orderly organization, and the vessels are often tortuous with irregular sizes, shapes, and branching patterns (10). The distribution of vessels is also heterogeneous, and the internal portions of the tumor can be less vascularized than the tumor-host interface (125). These abnormalities contribute to the unique characteristics of the TME, and they influence tumor growth, metastasis, and response to treatment.

Tumor angiogenesis and immune suppression frequently co-occur in response to various stimuli to facilitate the development and progression of tumors (126), and the abnormal vasculature influences the effectiveness of treatment profoundly. For example, the high permeability of the vessels leads to fluid accumulation and rising interstitial pressure, which can impede the delivery of drugs. The leaky nature of tumor vessels can facilitate the entry of cancer cells into the bloodstream, which increases the risk of metastasis (127). The uneven blood flow creates hypoxic and acidic areas within the tumor, which can promote tumor progression, aggravate the resistance to therapy, and nurture more aggressive cancer cell phenotypes. The abnormal tumor vasculature creates a barrier to immune cell infiltration, damaging the natural tumor surveillance by the immune system and reducing the effectiveness of immunotherapies. These conditions promote tumor growth, suppress the immune system, and contribute to treatment resistance, ultimately diminishing the effectiveness of chemotherapy, radiotherapy, and immunotherapy (123, 124).

## 5 Treating digestive system cancer with anti-angiogenic therapy and ICIs

### 5.1 Preclinical research

**Table 1** summarizes recent preclinical works that use ICIs and anti-angiogenic therapy for digestive system cancers.

**Table 1:** Preclinical studies of combined application of anti-angiogenic therapy and ICIs.

Antiangiogenic therapy	Immunotherapy	Tumor type	Therapeutic outcomes	Ref.
DC101 (anti-VEGFR2 mAb, 0.8 mg)	Anti-PD-1 mAb (clone RMP1-14, 0.25 mg)	Colon cancer	Inhibit angiogenesis Enhance T cell infiltration	(128)
DC101 (anti-VEGFR2 mAb, 40 mg/kg)	Anti-PD-L1 mAb (clone 10F.9G2, 10 mg/kg)	Pancreatic cancer Breast cancer Glioblastoma	Enhance antiangiogenic efficacy in pNET and BC Increase IFN-γ-expressing CD8^+^T and IFN-γ-expressing CD8^+^T cells Trigger the infiltration and activation of CTL Induce vessel normalization and HEV formation via LTβR	(129)
DC101 (anti-VEGFR2 mAb, 40 mg/kg)	Anti-PD-1 mAb (clone RMP-014, 10 mg/kg)	Hepatocellular carcinoma	Enhance the infiltration and activation of CTL Promote the polarization of TAM from M2 to M1 Reduce Treg and CCR2+ Reprogram the immune microenvironment	(130)
Lenvatinib (VEGFR1-3, FGFR1-4, PDGFRα, KIT, and REI, 10 mg/kg)	Anti-PD-1 mAb (10 mg/kg)	Hepatocellular carcinoma	Enhance the cytotoxic effects of T cells Reduce the proportion of monocytes and macrophages Regulate tumor vasculature normalization	(131)
Axitinib (VEGFR1-3, 25 mg/kg)	Anti-PD-1 mAb (10 mg/kg)	Lung cancer Colon cancer	Reduce mast cells and TAMs Reduce T-cell depletion Decrease the expression of inhibitory immune checkpoints on CD8^+^T cells	(132)
A2V (anti-VEGFA and ANGPT2)	Anti-PD-1 mAb (clone RMPI-14, 10 mg/kg)	Breast cancer Melanoma Pancreatic cancer	Impair tumor angiogenesis Increase tumor necrosis Induce the normalization of remaining vessels Activate tumor-infiltrating CD8^+^T cells Enhance tumor antigen presentation Promote T cell aggregation around blood vessels Enhance the anti-tumor activity of co-blocking ANGPT2 and VEGFA by anti-PD-1	(133)

VEGF and its corresponding receptors are widely regarded as the most prominent regulators of angiogenesis (25, 134). DC101 is an antiangiogenic monoclonal antibody that targets the VEGFR-2. Yasuda et al. demonstrated in a Colon-26 adenocarcinoma mouse model that the combination of an anti-PD-1 drug and DC101 exerts synergistic anti-tumor effects by activating tumor-infiltrating lymphocytes and inhibiting tumor neovascularization (128). Allen et al. found that DC101 and an anti-PD-L1 drug had synergistic effects in murine models of breast cancer (MMTV-PyMT), glioblastoma (NFpp10-GBM), and pancreatic neuroendocrine tumors (RT2-PNET), and the combination could increase IFN-γ+CD8+T cells, trigger tumor regression, and substantially improve survival rates (129). The underlying mechanism involved the formation of high endothelial venules (HEVs) and the activation of lymphotoxin β receptor (LTβR) signaling, and the antiangiogenic therapy improved the anti-PD-L1 treatment by generating intratumoral HEVs to facilitate the infiltration of CTLs and enhance their activities (130, 135). Shigeta et al. combined DC101 with an anti-PD-1 drug to markedly suppress tumor growth in a mouse model of HCC, which resulted in a twofold increase in the survival rate. The therapy works by increasing CTL infiltration and activation, altering the proportion of M2-TAMs, reducing Tregs and chemokines, and reshaping the TME (136).

Combination therapies targeting multiple angiogenic pathways are potentially more effective than therapies targeting VEGF alone, as when the VEGF signaling is inhibited, tumors may increase the expression of other growth factors (e.g., PDGF and FGF) to activate alternative angiogenic pathways (134). Deng et al. used the multi-target tyrosine kinase inhibitor (TKI) lenvatinib in conjunction with anti-PD-1 therapy in HCC mouse models and observed significant tumor reduction, higher remission rates, and the development of long-term immune memory (137). The combined treatment normalizes the tumor vasculature to enhance T cell cytotoxicity and reduce the proportion of monocytes and macrophages (131). Läubli et al. found that the TKI axitinib, when used alongside immunotherapy, significantly reduced tumor-promoting mast cells and TAMs, which substantially enhanced the survival rate (132).

When tumors become resistant to anti-VEGF therapy, the Ang-2/Tie-2 pathway can alternatively sustain tumor vascularization. The bispecific antibody A2V concurrently inhibits ANGPT2 and VEGFA to thus realize superior efficacy compared to monotherapy (138). It promotes vascular regression, tumor necrosis, and antigen presentation, while normalizing the remaining vessels and enhancing the infiltration and accumulation of CTLs. PD-1 blockade further enhances the antitumor efficacy of A2V (133, 138).

In summary, the combined potential of anti-angiogenic therapy and ICIs has been validated in various preclinical models. Considering other possible synergistic mechanisms, further exploration through expanded models is necessary (133, 139).

### 5.2 Clinical studies

**Table 2** summarizes 20 clinical trials, which cover esophageal squamous cell carcinoma (ESCC), gastric cancer (GC), gastroesophageal junction adenocarcinoma (GEJC), colorectal cancer (CRC), hepatocellular carcinoma (HCC), and biliary tract cancer (BTC). These trials used seven anti-angiogenic drugs, which are lenvatinib, bevacizumab anlotinib, apatinib, regorafenib, ramucirumab, and cabozantinib, and seven ICIs, which are pembrolizumab, atezolizumab, nivolumab, camrelizumab, sintilimab, durvalumab, and TQB2450. Four of the 20 trials also involved chemotherapy. All HCC trials except the JVDJ study included more than 100 patients, and other trials included 13 to 92 patients. The trials were examined as first-, second-, or third-line therapies.

**Table 2:** Clinical trials that use anti-angiogenic agents and ICIs to treat digestive system cancer.

Trial ID	Phase	Antiangiogenic drug	ICI drug^†^	Other Drugs	Results^†^	Ref.
Esophageal squamous cell carcinoma (ESCC)						
NCT04949256 (LEAP-014)	III	Lenvatinib	Pembrolizumab	Cisplatin + 5-FU or Cisplatin +Paclitaxel	On going	(148)
NCT05038813	II	Anlotinib	TQB2450	NA	ORR: 60.9%; DCR: 95.7%	(149)
NCT03736863 (CAP 02)	II	Apatinib	Camrelizumab	NA	ORR: 34.6%; DCR: 78.8% PFS: 6.8m; OS: 15.8m AEs: 44% (Grade ≥3)	(150)
Gastric cancer (GC) and gastroesophageal junction adenocarcinoma (GEJC)						
NCT03406871 (REGONIVO)^§^	I	Regorafenib	Nivolumab	NA	ORR: 44%; PFS: 5.6m	(151)
NCT04757363	II	Regorafenib	Nivolumab	FOLFOX	PFS at 6m: 71% OS at 12m: 85%	(152)
NCT03609359 (EPOC1706)	II	Lenvatinib	Pembrolizumab	NA	ORR: 69%; PFS: 7.1m	(153)
NCT02443324	I	Ramucirumab	Pembrolizumab	NA	ORR: 7%; DCR: 51% PFS: 2.6m; OS: 5.9m	(154)
NCT02572687 (JVDJ)^§^	I	Ramucirumab	Durvalumab	NA	ORR: 21% PFS: 2.6m; OS: 12.4m AEs: 37.9% (Grade ≥3)	(155)
Colorectal cancer (CRC)						
NCT04072198 (NIVACOR)	II	Bevacizumab	Nivolumab	FOLFOXIRI	ORR: 76.7%; DCR: 97.3% PFS: 10.1m	(156)
NCT04194359 (2020–552)	II	Bevacizumab	Sintilimab	CapeOX	ORR: 84%; DCR: 100%	(157)
NCT03406871 (REGONIVO)^§^	I	Regorafenib	Nivolumab	NA	ORR: 36%; PFS: 7.9m	(151)
NCT03797326 (LEAP-005)^§^	II	Lenvatinib	Pembrolizumab	NA	ORR: 22%; PFS: 7.5m	(158)
Hepatocellular carcinoma (HCC)						
NCT02572687 (JVDJ)^§^	I	Ramucirumab	Durvalumab	NA	ORR: 11% PFS: 4.4m; OS: 10.7m AEs: 42.9% (Grade ≥3)	(155)
NCT02715531 (GO30140)	I	Bevacizumab	Atezolizumab	NA	ORR: 36%; PFS: 5.6m	(159)
NCT03434379 (IMbrave150)	III	Bevacizumab	Atezolizumab	NA	ORR: 27%; PFS: 6.8m	(160)
NCT04102098 (IMbrave050)	III	Bevacizumab	Atezolizumab	NA	RFS: 78%	(161)
NCT03006926	I	Lenvatinib	Pembrolizumab	NA	ORR: 36%; DCR: 88% PFS: 8.6m; OS: 22.0m	(162)
NCT03713593 (LEAP-002)	III	Lenvatinib	Pembrolizumab	NA	PFS: 8.2m; OS: 21.2m	(163)
NCT03755791 (COSMIC-312)	III	Cabozantinib	Atezolizumab	NA	PFS: 6.8m; OS: 15.4m	(164)
NCT03764293 (RESCUE)	II	Apatinib	Camrelizumab	NA	ORR: 34.3%/23.8% DCR: 77.1%/75.8% PFS: 5.7m/5.5m	(165)
Biliary tract cancer (BTC)						
NCT03797326 (LEAP-005)^§^	II	Lenvatinib	Pembrolizumab	NA	ORR: 10%; DCR: 68% PFS: 6.1m; OS: 8.6m AEs: 48% (Grade ≥3)	(166)
JMA-IIA00436 (JCOG1808)	II	Lenvatinib	Nivolumab	NA	ORR: 9.4%; DCR: 53.1% PFS: 2.5m; OS: 6.4m	(167)
ChiCTR1900022003	II	Anlotinib	Sintilimab	NA	ORR: 30.0%; DCR: 95.0% PFS: 6.5m; OS: 12.3m AEs: 20.0% (Grade 3)	(168)

**^†^** ICI, immune checkpoint inhibitor; ORR, overall response rate; DCR, disease control rate; PFS, progression-free survival; OS, overall survival; RFS, recurrence-free survival; AEs, adverse events; NA, not applicable. ^§^ Trial appeared in more than one category.

Of the seven anti-angiogenic drugs, lenvatinib and bevacizumab were used in 6 and 5 of the 20 trials, respectively. Lenvatinib works synergistically with anti-PD-1 antibodies in the treatment of HCC (137). It inhibits multiple RTKs and disrupts the signaling pathways that promote tumor growth and angiogenesis by regulating VEGFRs, FGFRs, PDGFRs, etc. Bevacizumab is a humanized monoclonal antibody against VEGF-A that binds directly to VEGF and reduces tumor angiogenesis (140). It has been shown to improve the progression-free survival (PFS) in several cancers, including CRC, non-small cell lung cancer (NSCLC), renal cell carcinoma, glioblastoma, ovarian cancer, and cervical cancer.

Among the other anti-angiogenic drugs, apatinib is an oral tyrosine kinase inhibitor primarily used in the treatment of advanced or metastatic cancers, particularly GC (141). It works by selectively inhibiting VEGFR2 and promoting apoptosis, and it also targets other tyrosine kinases such as c-KIT and c-SRC (142). Anlotinib works by inhibiting VEGFRs, FGFRs, PDFGRs, and c-KIT, and it has shown promising results in treating NSCLC, soft tissue sarcoma, medullary thyroid carcinoma, metastatic renal cell carcinoma, and other solid tumors (143). Regorafenib works by inhibiting multiple protein kinases involved in tumor growth and angiogenesis, and it has been used to treat metastatic CRC, gastrointestinal stromal tumors, and HCC (144). Ramucirumab is a monoclonal antibody that targets VEGFR2 to inhibit VEGF-stimulated receptor phosphorylation and downstream signaling (145), and it has been used as various lines of treatment for GC, CRC, NSCLC, and HCC (146). Cabozantinib targets multiple tumor-associated RTKs, including the angiogenic growth factors VEGFR and MET, as well as the TAM family of kinases (TYRO3, AXL, MER). It has been used together with immunotherapy for advanced renal cell carcinoma and urothelial carcinoma (147).

#### 5.2.1 Esophageal squamous cell carcinoma

Three different regimens are currently under investigation for ESCC. In the phase III LEAP-014 study, Sun et al. explored the benefits of adding the anti-angiogenic agent lenvatinib to the existing first-line treatment of unresectable or metastatic ESCC (148). Preliminary results indicated that the combination of pembrolizumab, lenvatinib, and chemotherapy is safe and tolerable for previously untreated metastatic ESCC patients. Zhang et al. tested the combination of anlotinib with TQB2450 as a first-line therapy in advanced ESCC and found “encouraging efficacy and manageable adverse events”, with the overall response rate (ORR) and the disease control rate (DCR) being 60.9% and 95.7%, respectively (149). Meng et al. found that the combination of apatinib and camrelizumab, as a second-line treatment for advanced ESCC, had remarkable efficacy and maintained a manageable safety profile, giving 34.6% ORR and 93.2% DCR (150). The PFS was 6.8 months, and the overall survival (OS) was 15.8 months.

#### 5.2.2 Gastric cancer and gastroesophageal junction adenocarcinoma

Saeed et al. previously reviewed the combined use of ICIs and VEGF-targeting agents in treating advanced GC and GEJC (169). In a phase Ib trial (the REGONIVO study), Fukuoka et al. used regorafenib and nivolumab as a third-line treatment for GC and CRC (151). The ORR and PFS were, respectively, 44% and 5.6 months for the 25 gastric cancer patients, who all had unresectable recurrent solid tumors and were refractory or intolerant to standard chemotherapy. Regorafenib seemed to help to overcome the resistance to anti-PD-1 therapy, as in the seven patients with GC refractory to previous PD-1 therapy, the response to the regorafenib plus nivolumab regimen was achieved for three. The optimal daily dose of regorafenib was 80 mg, and further investigation in a larger cohort was deemed worthy. From a phase II trial, Cytryn et al. concluded that regorafenib can be safely combined with, and in fact strengthened, nivolumab and chemotherapy (the existing first-line standard) for HER2-negative metastatic esophagogastric cancer. Of all 35 patients being evaluated, the rate of PFS was 71% and 51% at 6 months and 12 months, and the rate of OS was 97% and 85% at 6 months and 12 months, respectively (152).

In the EPOC1706 study (a phase II trial), Kawazoe et al. evaluated the efficacy and safety of the combined use of lenvatinib and pembrolizumab in treating advanced GC/GEJC (153). The ORR was 69% (20 of 29 patients), and the PFS was 7.1 months. The combination gave a higher ORR than the single drugs, and the safety profile was manageable. In a phase Ia/b trial, Herbst et al. gave ramucirumab and pembrolizumab to patients with previously treated advanced GC/GEJC (154). Of the 41 enrolled participants, the ORR was 7%, the DCR was 51%, the PFS was 2.5 months, and the OS was 5.9 months. They argued that the dual inhibition of angiogenesis and immune checkpoint by the combination of VEGFR2 antagonist and PD-1 antagonist, with or without chemotherapy, was worthy of further exploration. Bang et al. conducted a phase Ia/b study, in which they used ramucirumab and durvalumab to treat advanced GC/GEJC (155). Of the 29 patients in the cohort, the ORR was 21%, the PFS was 2.6 months, and the OS was 12.4 months. The incidence of grade 3 or higher treatment-related AEs was 37.9%.

#### 5.2.3 Colorectal cancer

Immune therapy is now a standard primary treatment for metastatic CRC (mCRC) with high microsatellite instability. However, microsatellite stable (MSS) CRC, which accounts for around 95% of the CRC cases, does not respond well to ICI monotherapy (170). In the NIVACOR study, Damato et al. investigated the efficacy of combining Bevacizumab with nivolumab and FOLFOXIRI (fluorouracil + oxaliplatin + irinotecan) as a first-line treatment for RAS/BRAF mutated metastatic CRC patients (156). Of the 73 enrolled patients, the ORR was 76.7%, the DCR was 97.3%, and the PFS was 10.1 months. In a phase II trial, Yuan et al. combined sintilimab with CapeOx (oxaliplatin and capecitabine) and bevacizumab to treat patients with MSS and RAS-mutant mCRC (157). As of the data analysis date, the PFS was 17.9 and 9.79 months in the full analysis set and the per-protocol set, respectively. There were no grade 5 AEs during the study period. In view of the controllable safety and satisfying antitumor activity, a phase III clinical trial of this regimen is underway. The previously reviewed REGONIVO trial that used regorafenib with nivolumab for GC also covered CRC (151). Of the 25 CRC patients in the trial, the ORR and PFS were 36% and 7.9 months, respectively. In the LEAP-005 study, which is a phase II multi-cohort trial, Gomez-Roca et al. explored the efficacy of combining lenvatinib and pembrolizumab in patients who have already undergone treatment for solid tumors (158). In the CRC cohort, of the 32 enrolled patients, the ORR was 22%, and the OS was 7.5 months.

#### 5.2.4 Hepatocellular carcinoma

The trials for HCC are the most abundant in **Table 2**. In general, the combination of ICIs with anti-angiogenic agents has shown superiority compared to monotherapy, and multiple combination regimens appear as promising first-line treatments for advanced HCC.

The JVDJ study, which used Ramucirumab and Durvalumab, had different results for the HCC cohort compared to the GC/GEJC cohort (155). The ORR dropped to 11% and the OS dropped to 10.7 months, although the PFS increased to 4.4 months. It is the study with the smallest sample size (28 patients). The GO30140 study is a phase Ib clinical trial that demonstrated the safety and efficacy of the atezolizumab and bevacizumab regimen in HCC patients (159). The IMbrave150 study found that for patients with unresectable HCC, compared to standard treatment that used sorafenib, the atezolizumab/bevacizumab regimen exhibited significantly superior efficacy, as it improved the PFS from 4.3 months to 6.8 months and increased the OS at 12 months from 54.6% to 67.2% (160). The two groups had comparable incidence of Grade 3–4 AEs. The IMbrave050 study compared the atezolizumab/bevacizumab regimen to active surveillance in patients with resected or ablated high-risk HCC (161). Although the median RFS was not reached in either group, the atezolizumab/bevacizumab regimen increased the RFS event-free rates at 12 months from 65% to 78%.

The combination of lenvatinib and pembrolizumab has been examined in two trials. In a phase Ib study targeting patients with unresectable HCC, Finn et al. found that for the 100 patients being assessed, ORR was 36%, DCR was 88%, PFS was 8.6 months; and OS was 22.0 months (162). In the LEAP-002 study, which is a randomized, double-blind, phase III trial, Llovet et al. evaluated the benefits of adding pembrolizumab to lenvatinib in providing a first-line treatment for patients with unresectable hepatocellular carcinoma (163). Compared to the control group that used lenvatinib with placebo, the combination therapy group gave numerically superior OS (21.1 vs. 19.0 months) and PFS (8.2 vs. 8.0 months), but neither outcome reached the preset statistical significance. In the COSMIC-312 study, which is another phase III trial of a first-line systemic treatment for patients with advanced HCC, Kelley et al. found that compared to sorafenib, the combination of cabozantinib and atezolizumab significantly prolonged the PFS from 4.2 months to 6.8 months, but the improvement in OS was not statistically significant (164). Xu et al. tested the combination of camrelizumab and apatinib in the RESCUE study, which is a phase II trial involving patients with advanced HCC (171). The combination was given both as a first-line and a second-line treatment. The ORR was 34.3% and 22.5%, the DCR was 77.1% and 75.8%, and the PFS was 5.7 months and 5.5 months for the first- and second-line cohort, respectively.

#### 5.2.5 Biliary tract cancer

Villanueva et al. reported that for the BTC cohort in the abovementioned LEAP-005 study, which used lenvatinib/pembrolizumab as a second-line therapy, the ORR was 10%, the DCR was 68%, the PFS was 6.1 months, and the OS was 8.6 months (166). In the JCOG1808 study, Ueno et al. tested the nivolumab/lenvatinib combination as a second-line treatment of advanced BTC in 26 patients, and found that the ORR, DCR, PFS, and OS were 9.4%, 53.1%, 2.5 months, and 6.4 months, respectively (167). They suggested that the regimen had manageable safety but limited efficacy as a second-line treatment for patients with advanced BTC. In a phase II trial, Jin et al. tested sintilimab and anlotinib as a second-line therapy for patients who failed previous first-line systemic chemotherapy, and found that for the 20 patients in the full analysis set, the ORR, DCR, PFS, and OS were 30.0%, 95.0%, 6.5 months, and 12.3 months, respectively (168).

## 6 Conclusions and perspectives

Combining anti-angiogenic therapy with ICIs is an effective strategy to overcome tumor drug resistance, enhance treatment efficacy, and improve the prognosis of cancer patients. In tumors, the structures and functions of blood vessel are often compromised, and the resulting vascular abnormalities, including aberrant vessel dilation, irregular branching, lack of vascular wall integrity, etc., affect vascular permeability and hemodynamics. These problems contribute to the immune evasion and therapeutic resistance of tumors, as immune cells cannot work properly if they cannot efficiently infiltrate tissues via a healthy vascular system. In addition, tumor cells and other immune cells in the TME can also release inhibitory factors to cause EC dysfunction, thus further exacerbating vascular abnormalities. Normalizing the tumor blood vessels improves the infiltration of immune cells within the tumor and strengthens their cytotoxic effects, which ultimately increases the efficacy of immunotherapy. This review looked at the interactions between immune cells and tumor angiogenesis and summarized the latest advances in the combined application of anti-angiogenesis therapy and ICIs in treating digestive system tumors. Some failed and repeated trials were not included, and the ICIs were limited to anti-PD-1 and anti-PD-L1. The anti-angiogenesis therapies typically used humanized monoclonal antibodies or TKIs for vascular normalization within the TME, and ICIs were used for tumor immunotherapies.

The vascular-immune crosstalk strategy in cancer therapy also faces several challenges. First, the combined therapy may increase the toxicity of the treatment, and clinical research is needed to carefully balance safety and efficacy. Second, because tumors are highly heterogenous, it can be difficult to find biomarkers to comprehensively predict the outcomes of combined therapies. Selecting and validating predictive biomarkers is complicated because combined therapies involve multiple intertwining factors (e.g., tumor type, immune microenvironment, and angiogenesis status). Since the status of tumors and the immune system evolve during the treatment, the predictive biomarkers need to be capable of dynamic monitoring rather than only providing snapshots. The research on predictive biomarkers for combined therapies currently lacks standardized methods and evaluation systems, and it is hard to compare and validate findings across different studies. Third, it is essential to assess whether the effects from the combination are synergistic or merely additive. Studies are needed to identify the most effective sequence of administering ICIs and anti-angiogenic agents, as well as the optimal dosages and protocols for combination therapies. Fourth, it is crucial to accurately assess the vascular normalization window and determine appropriate start and endpoints in crafting treatment strategies. During vascular normalization, the structure and function of tumor vasculature temporarily approach normality. In this period, the vascular density and tortuosity of the tumor decrease, and pericytes are recruited to the existing tumor vasculature, thus improving tumor blood perfusion and reducing tissue hypoxia. With vascular normalization, anti-angiogenic therapy can be combined with radiotherapy, chemotherapy, or immunotherapy to enhance therapeutic effects by leveraging the improved vascular structure and TME. However, if the anti-angiogenic therapy is prolonged, the state of vascular normalization may disappear, thereby reactivating the “angiogenic switch.” Vascular homeostasis is regulated by multiple pro-angiogenic and anti-angiogenic factors. When these factors reach an equilibrium, the vascular system remains in a quiescent state, and endothelial cells cease proliferating. When pro-angiogenic signals predominate, angiogenesis is triggered, and in the TME, this process is known as the “angiogenic switch.” Once the “angiogenic switch” is activated, a large number of new blood vessels will form in the TME. They supply nutrients and oxygen to the tumor, thereby enhancing its invasiveness and metastatic potential. The window of vascular normalization is difficult to capture, and existing methods for detecting this window often suffer from poor reproducibility or operational complexity. Research is urgently needed to develop new biomarkers and reproducible detection methods for this window. A thorough understanding of the tumor biology and the effects of treatment is needed to maximize the treatment efficacy within the optimal time window. Finally, to deal with resistance, modulating pro-angiogenic signaling molecules such as PDGF, FGF-2, ANGPT, and APLN has become a hot research area. To better understand the working principles of combined therapies using anti-angiogenic drugs and ICIs, further research is still needed to explore the relevant mechanisms at the molecular level.
